# 4-(1*H*-Benzimidazol-2-ylmeth­oxy)-3-eth­oxy­benzaldehyde trihydrate

**DOI:** 10.1107/S1600536810027455

**Published:** 2010-07-17

**Authors:** Jerry P. Jasinski, Adam N. Braley, S. Samshuddin, B. Narayana, H. S. Yathirajan

**Affiliations:** a Department of Chemistry, Keene State College, 229 Main Street, Keene, NH 03435-2001, USA; b Department of Studies in Chemistry, Mangalore University, Mangalagangotri 574 199, India; c Department of Studies in Chemistry, University of Mysore, Manasagangotri, Mysore 570 006, India

## Abstract

In the title compound, C_17_H_16_N_2_O_3_·3H_2_O, the dihedral angle between the mean planes of the benzene and benzimidazole systems is 26.2 (3)°. These groups are slightly twisted around the eth­oxy­methane unit [C—C—O—C torsion angle = 177.64 (15)°]. The crystal packing is stabilized by N—H⋯O, O—H⋯N and O—H⋯O hydrogen-bond inter­actions involving the water mol­ecules. Weak π–π stacking inter­actions [centroid–centroid distances = 3.7943 (7), 3.6919 (13) and 3.7533 (14) Å] contribute to the mol­ecular stability.

## Related literature

For the biological activity of benzimidazoles, see: Pujar *et al.* (1988 ▶); Bouwman *et al.* 1990 ▶). For related structures, see: Madkour *et al.* (2006 ▶); Jian *et al.* (2003 ▶); Odabaşoğlu *et al.* (2007 ▶). For bond-length data, see: Allen *et al.* (1987 ▶).
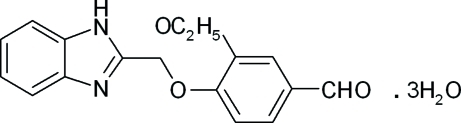


## Experimental

### 

#### Crystal data

C_17_H_16_N_2_O_3_·3H_2_O
*M* *_r_* = 350.37
Orthorhombic, 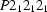

*a* = 7.3020 (15) Å
*b* = 9.3170 (19) Å
*c* = 25.950 (5) Å
*V* = 1765.4 (6) Å^3^
*Z* = 4
Cu *K*α radiation
μ = 0.84 mm^−1^
*T* = 100 K
0.51 × 0.45 × 0.39 mm

#### Data collection

Oxford Diffraction Xcalibur with a Ruby (Gemini CCD) detector diffractometer
Absorption correction: multi-scan (*CrysAlis RED*; Oxford Diffraction, 2007 ▶) *T*_min_ = 0.875, *T*_max_ = 1.000
4635 measured reflections
2130 independent reflections
2030 reflections with *I* > 2σ(*I*)
*R*_int_ = 0.019

#### Refinement

*R*[*F* ^2^ > 2σ(*F* ^2^)] = 0.035
*wR*(*F* ^2^) = 0.102
*S* = 1.06
2130 reflections
246 parameters
9 restraints
H atoms treated by a mixture of independent and constrained refinement
Δρ_max_ = 0.23 e Å^−3^
Δρ_min_ = −0.16 e Å^−3^

### 

Data collection: *CrysAlis PRO* (Oxford Diffraction, 2007 ▶); cell refinement: *CrysAlis RED* (Oxford Diffraction, 2007 ▶); data reduction: *CrysAlis RED*; program(s) used to solve structure: *SHELXS97* (Sheldrick, 2008 ▶); program(s) used to refine structure: *SHELXL97* (Sheldrick, 2008 ▶); molecular graphics: *SHELXTL* (Sheldrick, 2008 ▶); software used to prepare material for publication: *SHELXTL*.

## Supplementary Material

Crystal structure: contains datablocks global, I. DOI: 10.1107/S1600536810027455/fl2307sup1.cif

Structure factors: contains datablocks I. DOI: 10.1107/S1600536810027455/fl2307Isup2.hkl

Additional supplementary materials:  crystallographic information; 3D view; checkCIF report

## Figures and Tables

**Table 1:** Hydrogen-bond geometry (Å, °)

*D*—H⋯*A*	*D*—H	H⋯*A*	*D*⋯*A*	*D*—H⋯*A*
N2—H2*A*⋯O1*W*	0.86	1.96	2.822 (2)	175
O1*W*—H1*W*1⋯O3*W*^i^	0.84 (2)	1.87 (2)	2.703 (2)	171 (3)
O1*W*—H1*W*2⋯O1	0.83 (2)	2.09 (2)	2.911 (2)	170 (3)
O2*W*—H2*W*1⋯O2^ii^	0.82 (2)	2.04 (2)	2.855 (2)	178 (4)
O2*W*—H2*W*2⋯N1	0.81 (2)	2.04 (2)	2.836 (3)	169 (5)
O3*W*—H3*W*1⋯O2*W*	0.84 (2)	1.89 (2)	2.721 (3)	171 (4)
O3*W*—H3*W*2⋯O1*W*^iii^	0.83 (2)	2.05 (2)	2.870 (3)	171 (4)

Symmetry codes: (i) 
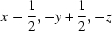
; (ii) 
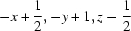
; (iii) 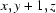.

**Fig. 1.**
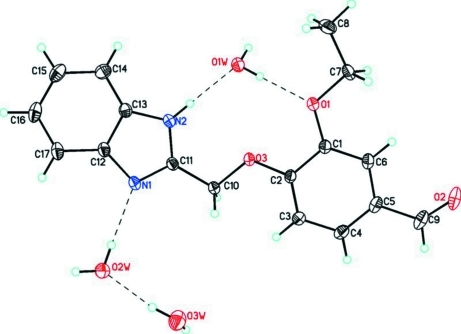
Molecular structure of C17H17N2O3, with 50% probability displacement ellipsoids. Dashed lines indicate O—H···N, O—H···O and N—H···O hydrogen bonds.

**Fig. 2.**
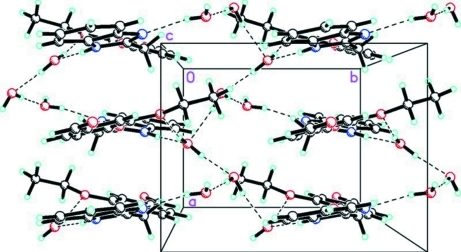
Packing diagram of the title compound viewed down the c axis. Dashed lines indicate O—H···N, O—H···O and N—H···O hydrogen bonds forming a two-dimensional chain along (011).

**Fig. 3.**
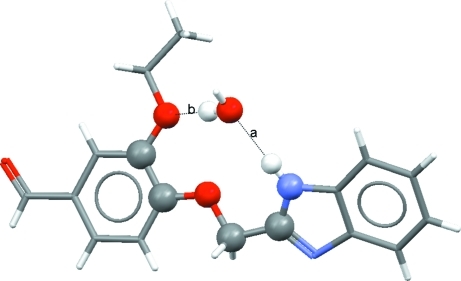
R22(10) graph-set motif for C17H17N2O3 involving lattice crystallized water molecules.

## supplementary crystallographic information

### Comment

The benzimidazole ring system and its related compounds play an important role in pharmaceutical and agricultural fields due to their broad spectrum of biological activities (Pujar *et al.*, 1988, Bouwman *et al.*, 1990). The synthesis of novel benzimidazole derivatives remains a main focus of medicinal research. Benzimidazoles are useful as insecticides, acaricides, nematocides, herbicides and other plant-protective agents in the field of pest control (Madkour *et al.*, 2006). In recent years, attention has increasingly been given to the synthesis of benzimidazole derivatives as a source of new antimicrobial agents. The crystal structures of some benzimidazole derivatives *viz*., 2-chloromethyl-1*H*-benzimidazole nitrate (Jian *et al.*, 2003) and 5-methoxy-1*H*-benzo[*d*]imidazole-2(3*H*)-thione (Odabaşoğlu *et al.*, 2007) have been reported. In view of the importance of benzimidazoles, the title compound, (I), ihas been synthesized and its crystal structure is reported here.

In (I) the dihedral angle between the mean planes of the benzene and benzimidazoles is 26.2 (6)° (Fig. 1). These groups are slightly twisted around the ethoxymethane structure (C2/C3/O3/C11 torsion angle = 177.64(15°). Bond distances and angles are in normal ranges (Allen *et al.*, 1987). Crystal packing is stabilized by O—H···O, O—H..N and N—H···O hydrogen bond interactions (Fig. 2, Table 1) involving lattice crystallized water molecules which form a *R*_2_^2^(10) graph-set motif (Fig. 3) and a two-dimensional chain along (001). Weak π-π stacking interactions (Table 2) contribute to molecular stability.

### Experimental

Ethyl vanillin (0.05 mol) was dissolved in 40 ml of ethanolic KOH (0.05 mol) and the solution was stirred for 1 h. 2-Chloromethyl-1*H*-benzimidazole (0.05 mole) was added with continuous stirring and refluxed for 5 h. The reaction mixture was cooled at room temperature, then poured into crushed ice. The solid product that separated out was filtered off and recrystallized using 1,4-dioxane. Single crystals were grown from ethanol slow evaporation method with a yield of 48%. (m.p.: 366 K). Analytical data: Found (Calculated): C %: 68.87(68.91); H%: 5.39 (5.44); N%: 9.40 (9.45).

### Refinement

The H atoms on the water molecules (H1W1, H1W2, H2W1, H2W2, H3W1, H3W2) were originally located in a difference Fourier and then their coordinates were allowed to refine with restraints to keep them in the range of 0.80 - 0.84 Å.  The remaining H atoms were then positioned geometrically and allowed to ride on their parent atoms with Atom—H lengths of 0.86Å (NH), 0.93 Å (CH), 0.97Å (CH_2_) or 0.96Å (CH_3_). Isotropic displacement parameters for these atoms were set to 1.5 times (OH), 1.2 times (NH), 1.2 (CH, CH_2_) or 1.5 (CH_3_) times *U*_eq_ of the parent atom. In the absence of anomalous scattering effects Friedel opposites were merged.

### Crystal data

**Table:**

C_17_H_16_N_2_O_3_·3H_2_O	*F*(000) = 744
*M**_r_* = 350.37	*D*_x_ = 1.318 Mg m^−^^3^
Orthorhombic, *P*2_1_2_1_2_1_	Cu *K*α radiation, λ = 1.54178 Å
Hall symbol: P 2ac 2ab	Cell parameters from 3976 reflections
*a* = 7.3020 (15) Å	θ = 4.7–77.2°
*b* = 9.3170 (19) Å	µ = 0.84 mm^−^^1^
*c* = 25.950 (5) Å	*T* = 100 K
*V* = 1765.4 (6) Å^3^	Block, colorless
*Z* = 4	0.51 × 0.45 × 0.39 mm

### Data collection

**Table:**

Oxford Diffraction Xcalibur with a Ruby (Gemini CCD) detector diffractometer	2130 independent reflections
Radiation source: Enhance (Cu) X-ray Source	2030 reflections with *I* > 2σ(*I*)
graphite	*R*_int_ = 0.019
Detector resolution: 10.5081 pixels mm^-1^	θ_max_ = 77.4°, θ_min_ = 5.9°
ω scans	*h* = −9→6
Absorption correction: multi-scan (*CrysAlis RED*; Oxford Diffraction, 2007)	*k* = −11→5
*T*_min_ = 0.875, *T*_max_ = 1.000	*l* = −32→32
4635 measured reflections	

### Refinement

**Table:**

Refinement on *F*^2^	Secondary atom site location: difference Fourier map
Least-squares matrix: full	Hydrogen site location: inferred from neighbouring sites
*R*[*F*^2^ > 2σ(*F*^2^)] = 0.035	H atoms treated by a mixture of independent and constrained refinement
*wR*(*F*^2^) = 0.102	*w* = 1/[σ^2^(*F*_o_^2^) + (0.0683*P*)^2^ + 0.161*P*] where *P* = (*F*_o_^2^ + 2*F*_c_^2^)/3
*S* = 1.06	(Δ/σ)_max_ < 0.001
2130 reflections	Δρ_max_ = 0.23 e Å^−^^3^
246 parameters	Δρ_min_ = −0.16 e Å^−^^3^
9 restraints	Extinction correction: *SHELXL97* (Sheldrick, 2008), Fc^*^=kFc[1+0.001xFc^2^λ^3^/sin(2θ)]^-1/4^
Primary atom site location: structure-invariant direct methods	Extinction coefficient: 0.018 (2)

### Special details

**Table:**

Experimental. In the absence of anomalous scattering effects Friedel opposites were merged
Geometry. All e.s.d.'s (except the e.s.d. in the dihedral angle between two l.s. planes) are estimated using the full covariance matrix. The cell e.s.d.'s are taken into account individually in the estimation of e.s.d.'s in distances, angles and torsion angles; correlations between e.s.d.'s in cell parameters are only used when they are defined by crystal symmetry. An approximate (isotropic) treatment of cell e.s.d.'s is used for estimating e.s.d.'s involving l.s. planes.
Refinement. Refinement of *F*^2^ against ALL reflections. The weighted *R*-factor *wR* and goodness of fit *S* are based on *F*^2^, conventional *R*-factors *R* are based on *F*, with *F* set to zero for negative *F*^2^. The threshold expression of *F*^2^ > σ(*F*^2^) is used only for calculating *R*-factors(gt) *etc*. and is not relevant to the choice of reflections for refinement. *R*-factors based on *F*^2^ are statistically about twice as large as those based on *F*, and *R*- factors based on ALL data will be even larger.

### Fractional atomic coordinates and isotropic or equivalent isotropic displacement parameters (Å^2^)

**Table:**

	*x*	*y*	*z*	*U*_iso_*/*U*_eq_	
O1	0.1649 (2)	0.05517 (14)	0.09028 (5)	0.0436 (3)	
N1	0.1237 (2)	0.36636 (17)	−0.10832 (5)	0.0413 (4)	
C1	0.1274 (2)	0.1882 (2)	0.10989 (6)	0.0380 (4)	
O2	0.1203 (3)	0.3165 (3)	0.26729 (6)	0.0857 (7)	
N2	0.0561 (2)	0.15054 (16)	−0.07628 (5)	0.0400 (3)	
H2A	0.0291	0.0857	−0.0539	0.048*	
C2	0.0862 (2)	0.2957 (2)	0.07342 (6)	0.0368 (4)	
O3	0.0958 (2)	0.25276 (13)	0.02315 (4)	0.0436 (3)	
C3	0.0434 (3)	0.4335 (2)	0.08910 (7)	0.0443 (4)	
H3	0.0156	0.5040	0.0650	0.053*	
C4	0.0424 (3)	0.4653 (2)	0.14156 (8)	0.0501 (5)	
H4	0.0121	0.5574	0.1524	0.060*	
C5	0.0857 (3)	0.3622 (3)	0.17741 (7)	0.0483 (5)	
C6	0.1279 (3)	0.2223 (2)	0.16162 (7)	0.0441 (4)	
H6	0.1561	0.1525	0.1860	0.053*	
C7	0.2193 (3)	−0.0543 (2)	0.12644 (8)	0.0484 (5)	
H7A	0.1208	−0.0732	0.1506	0.058*	
H7B	0.3260	−0.0231	0.1457	0.058*	
C8	0.2631 (4)	−0.1874 (2)	0.09646 (11)	0.0656 (7)	
H8A	0.1546	−0.2210	0.0794	0.098*	
H8B	0.3073	−0.2602	0.1195	0.098*	
H8C	0.3554	−0.1661	0.0713	0.098*	
C9	0.0857 (4)	0.3982 (3)	0.23244 (9)	0.0662 (7)	
H9	0.0565	0.4922	0.2413	0.079*	
C10	0.0577 (3)	0.35874 (19)	−0.01518 (6)	0.0408 (4)	
H10A	0.1400	0.4397	−0.0112	0.049*	
H10B	−0.0671	0.3930	−0.0115	0.049*	
C11	0.0832 (2)	0.29187 (19)	−0.06693 (6)	0.0369 (4)	
C12	0.1204 (3)	0.2666 (2)	−0.14792 (7)	0.0408 (4)	
C13	0.0800 (2)	0.1305 (2)	−0.12853 (7)	0.0411 (4)	
C14	0.0735 (3)	0.0092 (3)	−0.15962 (9)	0.0543 (5)	
H14	0.0505	−0.0814	−0.1461	0.065*	
C15	0.1030 (3)	0.0301 (3)	−0.21162 (9)	0.0644 (6)	
H15	0.1000	−0.0484	−0.2337	0.077*	
C16	0.1372 (4)	0.1668 (3)	−0.23188 (8)	0.0655 (7)	
H16	0.1525	0.1772	−0.2673	0.079*	
C17	0.1488 (3)	0.2858 (3)	−0.20093 (7)	0.0541 (5)	
H17	0.1746	0.3759	−0.2146	0.065*	
O1W	−0.0115 (3)	−0.06193 (16)	−0.00113 (5)	0.0539 (4)	
H1W1	−0.080 (4)	−0.128 (3)	0.0094 (11)	0.081*	
H1W2	0.025 (4)	−0.027 (3)	0.0264 (9)	0.081*	
O2W	0.2184 (4)	0.65334 (19)	−0.13309 (7)	0.0814 (7)	
H2W1	0.266 (5)	0.660 (4)	−0.1614 (10)	0.122*	
H2W2	0.206 (6)	0.569 (2)	−0.1259 (15)	0.122*	
O3W	0.2942 (3)	0.7793 (2)	−0.04092 (7)	0.0720 (5)	
H3W1	0.272 (5)	0.749 (4)	−0.0708 (9)	0.108*	
H3W2	0.198 (4)	0.820 (4)	−0.0315 (13)	0.108*	

### Atomic displacement parameters (Å^2^)

**Table:**

	*U*^11^	*U*^22^	*U*^33^	*U*^12^	*U*^13^	*U*^23^
O1	0.0604 (8)	0.0383 (6)	0.0320 (5)	0.0041 (6)	−0.0044 (6)	0.0040 (5)
N1	0.0509 (8)	0.0405 (7)	0.0325 (7)	0.0022 (7)	0.0009 (6)	0.0080 (6)
C1	0.0385 (8)	0.0428 (9)	0.0326 (8)	−0.0030 (7)	−0.0005 (7)	0.0004 (7)
O2	0.1008 (14)	0.1227 (17)	0.0337 (7)	−0.0013 (15)	−0.0111 (8)	−0.0134 (10)
N2	0.0478 (8)	0.0384 (7)	0.0337 (7)	−0.0014 (7)	0.0012 (6)	0.0067 (6)
C2	0.0405 (8)	0.0409 (8)	0.0291 (7)	−0.0008 (7)	0.0006 (6)	−0.0013 (6)
O3	0.0653 (8)	0.0388 (6)	0.0269 (5)	0.0073 (6)	−0.0005 (5)	0.0022 (5)
C3	0.0515 (9)	0.0413 (9)	0.0401 (8)	0.0015 (8)	0.0024 (8)	−0.0028 (7)
C4	0.0540 (10)	0.0490 (10)	0.0473 (10)	−0.0017 (9)	0.0058 (9)	−0.0143 (9)
C5	0.0459 (9)	0.0633 (12)	0.0355 (8)	−0.0081 (10)	0.0007 (7)	−0.0116 (8)
C6	0.0465 (9)	0.0549 (10)	0.0309 (8)	−0.0040 (9)	−0.0015 (7)	0.0009 (8)
C7	0.0557 (10)	0.0499 (10)	0.0398 (8)	−0.0017 (9)	−0.0057 (8)	0.0154 (8)
C8	0.0841 (16)	0.0432 (10)	0.0696 (14)	0.0093 (11)	−0.0202 (13)	0.0082 (10)
C9	0.0687 (14)	0.0878 (18)	0.0420 (11)	−0.0078 (14)	−0.0006 (10)	−0.0233 (12)
C10	0.0529 (10)	0.0376 (8)	0.0319 (7)	0.0034 (8)	−0.0003 (7)	0.0045 (7)
C11	0.0403 (8)	0.0375 (8)	0.0330 (7)	0.0029 (7)	−0.0005 (7)	0.0055 (6)
C12	0.0416 (8)	0.0479 (9)	0.0330 (8)	0.0046 (8)	−0.0010 (7)	0.0054 (7)
C13	0.0386 (8)	0.0462 (9)	0.0385 (8)	0.0004 (8)	−0.0029 (7)	0.0025 (7)
C14	0.0540 (11)	0.0515 (11)	0.0574 (11)	−0.0048 (10)	−0.0042 (9)	−0.0096 (9)
C15	0.0611 (12)	0.0792 (16)	0.0529 (12)	−0.0008 (13)	−0.0085 (10)	−0.0251 (12)
C16	0.0672 (14)	0.0958 (18)	0.0333 (9)	0.0059 (15)	−0.0025 (9)	−0.0080 (11)
C17	0.0602 (12)	0.0686 (13)	0.0335 (8)	0.0038 (11)	0.0006 (8)	0.0091 (9)
O1W	0.0762 (10)	0.0454 (7)	0.0400 (6)	−0.0085 (8)	−0.0039 (7)	0.0033 (6)
O2W	0.1387 (19)	0.0502 (9)	0.0554 (9)	−0.0111 (12)	0.0363 (11)	−0.0038 (7)
O3W	0.0808 (11)	0.0771 (11)	0.0581 (9)	0.0230 (11)	−0.0052 (9)	−0.0030 (9)

### Geometric parameters (Å, °)

**Table:**

O1—C1	1.367 (2)	C8—H8A	0.9600
O1—C7	1.442 (2)	C8—H8B	0.9600
N1—C11	1.313 (2)	C8—H8C	0.9600
N1—C12	1.386 (2)	C9—H9	0.9300
C1—C6	1.380 (2)	C10—C11	1.492 (2)
C1—C2	1.410 (2)	C10—H10A	0.9700
O2—C9	1.209 (4)	C10—H10B	0.9700
N2—C11	1.354 (2)	C12—C13	1.397 (3)
N2—C13	1.380 (2)	C12—C17	1.402 (2)
N2—H2A	0.8600	C13—C14	1.389 (3)
C2—O3	1.3660 (19)	C14—C15	1.380 (3)
C2—C3	1.383 (3)	C14—H14	0.9300
O3—C10	1.429 (2)	C15—C16	1.400 (4)
C3—C4	1.393 (3)	C15—H15	0.9300
C3—H3	0.9300	C16—C17	1.372 (4)
C4—C5	1.374 (3)	C16—H16	0.9300
C4—H4	0.9300	C17—H17	0.9300
C5—C6	1.400 (3)	O1W—H1W1	0.840 (17)
C5—C9	1.467 (3)	O1W—H1W2	0.829 (17)
C6—H6	0.9300	O2W—H2W1	0.815 (18)
C7—C8	1.498 (3)	O2W—H2W2	0.809 (19)
C7—H7A	0.9700	O3W—H3W1	0.842 (18)
C7—H7B	0.9700	O3W—H3W2	0.832 (18)
			
C1—O1—C7	117.02 (14)	H8B—C8—H8C	109.5
C11—N1—C12	104.39 (15)	O2—C9—C5	125.8 (3)
O1—C1—C6	124.79 (17)	O2—C9—H9	117.1
O1—C1—C2	115.88 (14)	C5—C9—H9	117.1
C6—C1—C2	119.33 (17)	O3—C10—C11	108.28 (14)
C11—N2—C13	106.84 (15)	O3—C10—H10A	110.0
C11—N2—H2A	126.6	C11—C10—H10A	110.0
C13—N2—H2A	126.6	O3—C10—H10B	110.0
O3—C2—C3	124.35 (16)	C11—C10—H10B	110.0
O3—C2—C1	114.97 (15)	H10A—C10—H10B	108.4
C3—C2—C1	120.67 (16)	N1—C11—N2	113.62 (16)
C2—O3—C10	116.91 (14)	N1—C11—C10	122.95 (16)
C2—C3—C4	119.09 (18)	N2—C11—C10	123.31 (15)
C2—C3—H3	120.5	N1—C12—C13	110.22 (15)
C4—C3—H3	120.5	N1—C12—C17	129.7 (2)
C5—C4—C3	120.76 (19)	C13—C12—C17	120.0 (2)
C5—C4—H4	119.6	N2—C13—C14	132.61 (19)
C3—C4—H4	119.6	N2—C13—C12	104.93 (16)
C4—C5—C6	120.21 (17)	C14—C13—C12	122.45 (17)
C4—C5—C9	119.9 (2)	C15—C14—C13	116.6 (2)
C6—C5—C9	119.8 (2)	C15—C14—H14	121.7
C1—C6—C5	119.92 (19)	C13—C14—H14	121.7
C1—C6—H6	120.0	C14—C15—C16	121.5 (2)
C5—C6—H6	120.0	C14—C15—H15	119.2
O1—C7—C8	107.84 (16)	C16—C15—H15	119.2
O1—C7—H7A	110.1	C17—C16—C15	121.76 (19)
C8—C7—H7A	110.1	C17—C16—H16	119.1
O1—C7—H7B	110.1	C15—C16—H16	119.1
C8—C7—H7B	110.1	C16—C17—C12	117.5 (2)
H7A—C7—H7B	108.5	C16—C17—H17	121.2
C7—C8—H8A	109.5	C12—C17—H17	121.2
C7—C8—H8B	109.5	H1W1—O1W—H1W2	102 (2)
H8A—C8—H8B	109.5	H2W1—O2W—H2W2	109 (3)
C7—C8—H8C	109.5	H3W1—O3W—H3W2	105 (3)
H8A—C8—H8C	109.5		
			
C7—O1—C1—C6	−3.9 (3)	C12—N1—C11—N2	0.8 (2)
C7—O1—C1—C2	175.99 (16)	C12—N1—C11—C10	−175.35 (17)
O1—C1—C2—O3	−2.2 (2)	C13—N2—C11—N1	−0.3 (2)
C6—C1—C2—O3	177.71 (16)	C13—N2—C11—C10	175.87 (16)
O1—C1—C2—C3	178.95 (16)	O3—C10—C11—N1	−154.73 (17)
C6—C1—C2—C3	−1.1 (3)	O3—C10—C11—N2	29.4 (2)
C3—C2—O3—C10	−0.3 (3)	C11—N1—C12—C13	−1.1 (2)
C1—C2—O3—C10	−179.14 (16)	C11—N1—C12—C17	178.2 (2)
O3—C2—C3—C4	−178.37 (18)	C11—N2—C13—C14	178.3 (2)
C1—C2—C3—C4	0.4 (3)	C11—N2—C13—C12	−0.4 (2)
C2—C3—C4—C5	0.9 (3)	N1—C12—C13—N2	0.9 (2)
C3—C4—C5—C6	−1.3 (3)	C17—C12—C13—N2	−178.49 (18)
C3—C4—C5—C9	179.4 (2)	N1—C12—C13—C14	−177.95 (18)
O1—C1—C6—C5	−179.41 (18)	C17—C12—C13—C14	2.7 (3)
C2—C1—C6—C5	0.7 (3)	N2—C13—C14—C15	179.3 (2)
C4—C5—C6—C1	0.5 (3)	C12—C13—C14—C15	−2.2 (3)
C9—C5—C6—C1	179.83 (19)	C13—C14—C15—C16	−0.1 (4)
C1—O1—C7—C8	−176.90 (18)	C14—C15—C16—C17	2.1 (4)
C4—C5—C9—O2	179.5 (3)	C15—C16—C17—C12	−1.6 (4)
C6—C5—C9—O2	0.2 (4)	N1—C12—C17—C16	−179.9 (2)
C2—O3—C10—C11	177.64 (15)	C13—C12—C17—C16	−0.7 (3)

### Hydrogen-bond geometry (Å, °)

**Table:**

*D*—H···*A*	*D*—H	H···*A*	*D*···*A*	*D*—H···*A*
N2—H2A···O1W	0.86	1.96	2.822 (2)	175
O1W—H1W1···O3W^i^	0.84 (2)	1.87 (2)	2.703 (2)	171 (3)
O1W—H1W2···O1	0.83 (2)	2.09 (2)	2.911 (2)	170 (3)
O2W—H2W1···O2^ii^	0.82 (2)	2.04 (2)	2.855 (2)	178 (4)
O2W—H2W2···N1	0.81 (2)	2.04 (2)	2.836 (3)	169 (5)
O3W—H3W1···O2W	0.84 (2)	1.89 (2)	2.721 (3)	171 (4)
O3W—H3W2···O1W^iii^	0.83 (2)	2.05 (2)	2.870 (3)	171 (4)

Symmetry codes: (i) *x*−1/2, −*y*+1/2, −*z*; (ii) −*x*+1/2, −*y*+1, *z*−1/2; (iii) *x*, *y*+1, *z*.

### Table 2 Cg···Cg π-stacking interactions

Cg1 is the centroid of ring C11/N1/C12/C13/N2; Cg2 is the centroid of the ring C1–C6; Cg3 is the centroid of ring C12-C17.

**Table:**

	*Cg*I···*Cg*J	CgI···Perp (Å)	CgJ···Perp (Å)
Cg1···Cg2^i^	3.7943 (7)	3.4481 (7)	-3.5681 (8)
Cg1···Cg2^ii^	3.6919 (13)	-3.61041 (7)	3.5404 (8)
Cg2···Cg3^i^	3.7533 (14)	3.5487 (8)	3.3763 (9)

Symmetry codes: (i) -1/2-x, 1/2-y, -z; (ii) 1/2+x, 1/2-y,-z.
